# Structural genomics of infectious disease drug targets: the SSGCID

**DOI:** 10.1107/S1744309111029204

**Published:** 2011-08-13

**Authors:** Robin Stacy, Darren W. Begley, Isabelle Phan, Bart L. Staker, Wesley C. Van Voorhis, Gabriele Varani, Garry W. Buchko, Lance J. Stewart, Peter J. Myler

**Affiliations:** a Seattle Structural Genomics Center for Infectious Disease, USA; b Seattle Biomedical Research Institute, 307 Westlake Avenue North, Suite 500, Seattle, WA 98109-5219, USA; c Emerald BioStructures, 7869 NE Day Road West, Bainbridge Island, WA 98110, USA; d Department of Medicine, Division of Allergy and Infectious Diseases, University of Washington, Box 357185, Seattle, WA 98195, USA; e Departments of Chemistry and Biochemistry, University of Washington, Box 351700, Seattle, WA 98185, USA; f Biological Sciences Division, Pacific Northwest National Laboratory, Richland, WA 99354, USA; g Departments of Global Health and Medical Education and Biomedical Informatics, University of Washington, Box 357238, Seattle, WA 98195, USA

**Keywords:** SSGCID, structural genomics, structure-based drug design, infectious diseases, pathogens, emerging and re-emerging diseases

## Abstract

The Seattle Structural Genomics Center for Infectious Disease (SSGCID) is a consortium of researchers at Seattle BioMed, Emerald BioStructures, the University of Washington and Pacific Northwest National Laboratory that was established to apply structural genomics approaches to drug targets from infectious disease organisms. The SSGCID is currently funded over a five-year period by the National Institute of Allergy and Infectious Diseases (NIAID) to determine the three-dimensional structures of 400 proteins from a variety of Category A, B and C pathogens. Target selection engages the infectious disease research and drug-therapy communities to identify drug targets, essential enzymes, virulence factors and vaccine candidates of biomedical relevance to combat infectious diseases. The protein-expression systems, purified proteins, ligand screens and three-dimensional structures produced by SSGCID con­stitute a valuable resource for drug-discovery research, all of which is made freely available to the greater scientific community. This issue of *Acta Crystallographica Section F*, entirely devoted to the work of the SSGCID, covers the details of the high-throughput pipeline and presents a series of structures from a broad array of pathogenic organisms. Here, a background is provided on the structural genomics of infectious disease, the essential components of the SSGCID pipeline are discussed and a survey of progress to date is presented.

## Abstract 2

An introduction and overview of the focus, goals and overall mission of the Seattle Structural Genomics Center for Infectious Disease (SSGCID) is given.

## 1. Structural genomics of infectious disease: a short history   

Over the past decade, structure-based drug design has played an increasingly important role in drug development. To this end, considerable effort and resources have been devoted to solving important protein structures from human pathogens (Van Voorhis *et al.*, 2009 ▶), leading to the establishment of several structural genomics consortia. The first group, the Structural Genomics of Pathogenic Protozoa Consortium (SGPP; http://www.sgpp.org/) solved 70 structures of proteins from pathogenic protozoa, developing methods and insights that were subsequently used by the Medical Structural Genomics of Pathogenic Protozoa Project (MSGPP; http://www.msgpp.org/) to develop novel antiprotozoan drugs (Fan *et al.*, 2008 ▶). The Tuberculosis Structural Genomics Consortium (TBSGC; http://www.webtb.org/) unifies core facilities to service more than 100 individual laboratories and focuses on the structure determination of metabolic and other functionally important proteins from *Mycobacterium tuberculosis* to aid in drug discovery (Goulding *et al.*, 2002 ▶; Terwilliger *et al.*, 2003 ▶). Although the Structural Genomics Consortium (SGC; http://www.sgc.utoronto.ca/) focuses heavily on human disease proteins, this group also studies kinases, cylophilins, ubiquitin-conjugating enzymes and a number of salvage and biosynthesis pathways from eukaryotic parasites, including trypanosomes, *Plasmodium falciparum* and their apicomplexan orthologues (Gileadi *et al.*, 2007 ▶; Bochkarev & Tempel, 2008 ▶). The Viral Infection Structural Proteomics (VISP) Center (http://visp.scripps.edu/default.aspx) solves protein structures from SARS-CoV, influenza, herpesviruses and flaviviruses. In addition, the biological community has nominated a number of microbial targets for structure solution by the Protein Structure Initiative (PSI) network (http://www.sbkb.org/). In particular, the Midwest Center for Structural Genomics (MCSG; http://www.mcsg.anl.gov/) addresses proteins related to pathogenesis, metabolism, host interactions and disease (Lee *et al.*, 2011 ▶). By September 2007, these cumulative efforts and those from individual research laboratories had resulted in over 3700 Protein Data Bank entries for proteins from pathogenic organisms on the NIAID Category A, B and C Priority Pathogens list, excluding *Escherichia coli*.

In late 2007, the National Institute of Allergy and Infectious Diseases (NIAID) provided funding to both the Seattle Structural Genomics Center for Infectious Disease (SSGCID; http://www.ssgcid.org) and the Center for Structural Genomics of Infectious Diseases (CSGID; http://www.csgid.org/) to solve protein structures from potential bioterrorism agents and emerging and re-emerging infectious disease organisms (Myler *et al.*, 2009 ▶; Anderson, 2009 ▶). These organisms include 31 different genera of bacteria, eukaryotes and viruses, which have been divided between the two centers. In striving to meet the needs of infectious disease researchers within the greater scientific community, the SSGCID interacts heavily with academic collaborators to solicit target nominations and to freely provide for them structural data, as well as clones, purified proteins and other laboratory materials, for primary research purposes (Myler *et al.*, 2009 ▶). The work of the SSGCID, the CSGID and other specialized centers represents an increased focus within the National Institutes of Health to address a broad range of biological problems relevant to particular sectors of scientific investigation. Thus, the SSGCID represents a unique structural biology resource for researchers focused on the discovery and development of novel cures or treatments for infectious diseases.

## 2. SSGCID: the Seattle Structural Genomics Center for Infectious Disease   

The SSGCID consortium consists of team members from four institutions in the Pacific Northwest of the United States: Seattle Biomedical Research Institute (Seattle BioMed), Emerald Bio­Structures (EmBios, formerly deCODE bioStructures), the University of Washington (UW) and Pacific Northwest National Laboratory (PNNL). The consortium is advised by an external panel of experts, and a Target Selection Board reviews targets selected by the con­sortium itself prior to submission to NIAID for approval. Community requests for novel protein structures are reviewed and approved by NIAID with the highest priority prior to entry into the SSGCID structure-determination pipeline. The SSGCID workflow is divided into several major activities: Target Selection, Cloning and Expression Testing, Protein Production, Crystallization, X-ray and NMR Data Collection, Structure Solution, and Project and Data Management. The first will be described briefly below, with the remaining activities explored in more detail in the Laboratory, Crystallization and Structure Communications contained in this volume of *Acta Crystallographica Section F*.

### 2.1. Target selection   

SSGCID focuses its structure-determination efforts on eight genera of bacteria (*Bartonella*, *Brucella*, *Ehrlichia*, *Anaplasma*, *Rickettsia*, *Burkholderia*, *Mycobacterium* and *Borrelia)*, nine species of eukaryotic pathogens (*Acanthamoeba*, *Babesia*, *Cryptosporidium*, *Cyclospora*, *Toxoplasma*, *Giardia*, *Entamoeba*, *Coccidioides* and *Encephalitozoon*), 13 negative-strand RNA viruses (Marburg virus, Ebola-like virus, influenza A, B and C viruses, *Arenavirus*, *Hantavirus*, *Henipavirus*, *Lyssavirus*, *Nairovirus*, *Orthobunyavirus*, *Phlebovirus* and *Rubulavirus*) and one single-stranded DNA virus (*Erythrovirus*). To date, a total of 7564 targets from 65 species within 24 genera have been validated and approved for the SSGCID pipeline. At the outset of this project, the SSGCID bioinformatics team selected several thousand proteins thought to represent drug targets in SSGCID target organisms since they play key roles in, or were identified as markers of, infectivity, reproduction, growth and drug resistance. For bacterial and eukaryotic pathogens, initial target selections were made by identifying homologues to potential drug targets in a single ‘representative’ species/strain from each genus based on similarity to targets in the DrugBank database (http://www.drugbank.ca/). Additional details covering the initial target-selection approaches, including the bioinformatic filters utilized, have been described previously (Myler *et al.*, 2009 ▶). Target selection at SSGCID also includes rescue attempts for failed targets by selecting orthologues or paralogues in other species within the NIAID-approved genera. This has been performed for eight *Mycobacterium* genomes (*M. abscessus*, *M. avium*, *M. bovis*, *M. leprae*, *M. marinum*, *M. paratuberculosis*, *M. smegmatis* and *M. thermoresistibile*) in order to characterize homologues of *M. tuberculosis* targets which had failed at some stage within the SSGCID pipeline. We have also used a bioinformatic approach that utilizes a statistical classification algorithm (Cadag *et al.*, 2008 ▶) to identify proteins predicted to be associated with virulence and/or pathogenesis. Viral genomes contain substantially fewer protein-coding genes than bacterial or eukaryotic pathogens and therefore a different approach was adopted for target selection in these genera. Following the recommendation of the viral research community, we focused on two potential drug targets involved in viral replication: nucleoprotein (N) and RNA-dependent RNA polymerase (L). Orthologues of these targets were selected from several genera, species or strains for each virus family. This strategy has already been applied to the *Bunyaviridae*, *Paramyxoviridae* and *Rhabdoviridae* families and will be extended to *Arenaviridae*, *Orthomyxoviridae* and *Parvoviridae*.

As awareness of the SSGCID has permeated the scientific community, the pipeline of internally selected targets has become supplemented with increasing numbers of targets requested by community researchers. Interaction with collaborative researchers continues to influence the SSGCID pipeline, leading to the selection of entire biological pathways that appear to be essential in one or more pathogenic organisms. For instance, several community requests included all seven enzymes of the methylerythritol phosphate (MEP) pathway for isoprenoid biosynthesis from a number of bacterial and protozoan species. Enzymes in this pathway have been demonstrated to be essential in malaria, tuberculosis and a variety of other protozoan and bacterial organisms, in contrast to the mevalonate-dependent pathway that is present in humans (Rohmer *et al.*, 1993 ▶; Jomaa *et al.*, 1999 ▶; Eisenreich *et al.*, 2004 ▶; Hunter, 2007 ▶). SSGCID has also expanded beyond proteins to include a small number of noncoding RNA molecules, such as bacterial thi-box, SAM-II and preQ1 riboswitches, for structure determination. This work includes efforts to determine the structure of a ligand-bound viral RNA complex identified by the UW-NMR group together with a community collaborator. Such noncanonical macromolecular com­plexes represent ground-breaking efforts to expand the range of biological targets amenable to drug targeting and represent efforts to better understand biological mechanisms which are essential for the growth and proliferation of infectious disease organisms.

### 2.2. Structure-determination pipeline   

**Figure 1:**
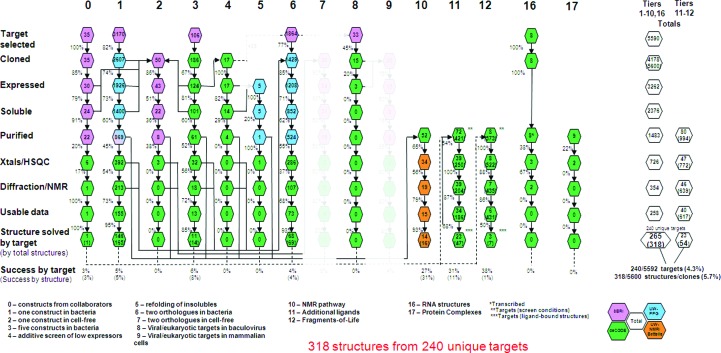
The SSGCID pipeline. A 17-tiered serial escalation approach is utilized by the SSGCID, with activities performed at Seattle BioMed (pink), UW-PPG (blue), Emerald BioStructures (green) and UW-NMR or PNNL (orange). Each Tier utilizes the approach described at the bottom of the figure. The numbers in the hexagons indicate the numbers of targets which have successfully passed through each step of the pipeline.

The methodologies used within SSGCID for cloning, expression testing, protein production, crystallization and structure determination have been described previously (Myler *et al.*, 2009 ▶), with further detail and recent improvements described in the accompanying articles. Most targets entering the SSGCID pipeline (Fig. 1 ▶) are cloned into the SSGCID standard bacterial expression vector (pAVA0421) by PCR amplification from genomic DNA or cDNA (Tier 1). A relatively small percentage of target plasmids come directly from collaborators (Tier 0) or are cloned using gene synthesis (Tier 3). Multiple rescue pathways (Tiers 2–9) allow increased success in either expression or purification with any target and are prioritized for community-request targets. With purified protein in hand, crystallization trials are set up in standard screens using two 96-condition sparse-matrix screens and two 96 grid-condition screens. In addition, a substantial number of SSGCID proteins have been screened using the Microcapillary Protein Crystallization System (MPCS) developed by the Protein Structure Initiative (PSI) ATCG3D technology center (Gerdts *et al.*, 2008 ▶, 2010 ▶). High-priority small-molecular-weight targets that fail to crystallize are selected for NMR-based analysis and structure determination at PNNL or UW-NMR (Tier 10). For every unique macromolecular structure solved by the SSGCID, model coordinates and structure factors are deposited in the Protein Data Bank (http://www.pdb.org) to provide the broadest possible public access (Berman *et al.*, 2000 ▶, 2003 ▶). Every apoprotein structure successfully solved is then bioinformatically processed in an attempt to find putative cofactors, inhibitors or other ligands for cocrystallization trials. This process employs biochemical searches for enzyme-reaction substrates and cofactors by mining databases that contain ligand or potential inhibitor interactions. Chemical abstract service (CAS) numbers or other identifiers are then used to query vendor databases for ordering. In addition to targeted ligand-complex studies, SSGCID annually selects a small number of high-impact targets for a complete Fragments-of-Life library screen (Tier 12; Begley, Davies *et al.*, 2011 ▶; Davies *et al.*, 2009 ▶). This library now contains over 2000 metabolites, their bioisosteres and other small molecules designed to mimic compounds found within the natural metabolome. Studies with high-priority SSGCID targets have led to the refinement of fragment-based screening techniques by NMR spectroscopy (Begley, Davies *et al.*, 2011 ▶) and X-ray crystallography (Begley, Hartley *et al.*, 2011 ▶). Lastly, RNA targets enter Tier 16 and protein complexes enter Tier 17, with special protocols adapted for pipeline production of these classes of macromolecules (Fig. 1 ▶).

### 2.3. Target status and success rates   

**Figure 2:**
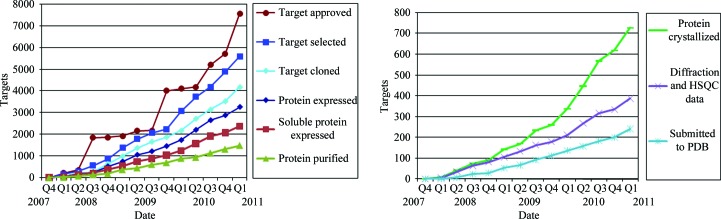
Cumulative status at key steps of targets in the SSGCID pipeline.

To date, 7564 targets have been approved for entry into the SSGCID structure-determination pipeline (Fig. 2 ▶), including 1384 which were either nominated or claimed by the scientific community. The SSGCID has cloned a total of 4178 targets, of which 2376 have expressed soluble protein and 1483 have been prepared to high purity from cell extracts. Of these, 726 have yielded crystals amenable to X-­ray diffraction, resulting in X-ray structures for 226 targets. Heteronuclear single-quantum coherence (HSQC) spectra have been acquired for an additional 34 targets, 14 of which have led to complete solution-state structure determination by standard protein-based NMR experiments. These 240 different targets have led to 318 structures being submitted to the PDB, of which 112 (from 70 targets) contained bound ligands. The overall structure-determination success rate for the 3383 bacterial, 758 eukaryotic and 37 viral targets cloned by SSGCID currently stands at ∼6%, but the success rate varies considerably (from 1 to 18%) between genera (Table 1 ▶). While the solubility rate is surprisingly similar for prokaryotes and eukaryotes (57 *versus* 56%, respectively), some bacteria (*Borrelia* and *Rickettsia*) and a number of eukaryotes (*Cryptosporidium*, *Encephalitozoon*, *Entamoeba*, *Giardia* and *Toxoplasma*) perform relatively poorly. Interestingly, the purification success rates are lower for prokaryotes (60%) than eukaryotes (71%); this may be the result of a large number of soluble *Burkholderia* targets having not yet been purified. Crystallization and diffraction rates from eukaryotic proteins are lower (44% and 43%, respectively) than those from prokaryotes (51% and 51%, respectively). However, once high-quality diffraction data have been obtained the rates of structure solution are similar for both kingdoms.

## 3. Community outreach   

### 3.1. Target nomination   

**Figure 3:**
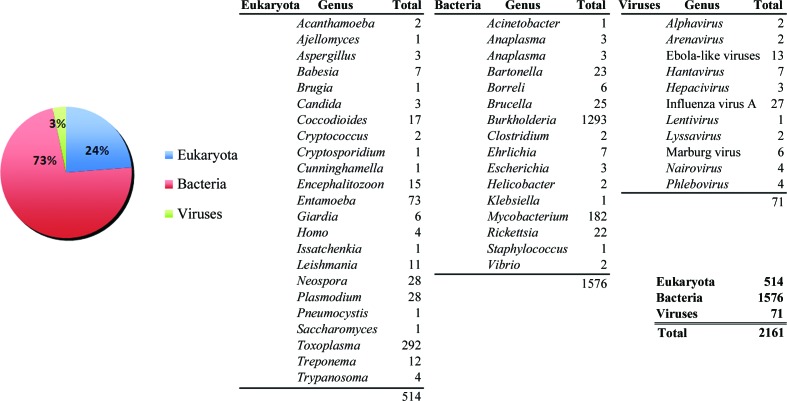
Summary of community-request targets by kingdom (pie chart) and genus (tables).

The most important mandate for the SSGCID is to provide three-dimensional protein structure-determination services to the scientific community at no charge. Target nominations from requestors may be submitted online (at http://www.ssgcid.org/home/Community.asp) and such nominations are given the highest priority in the SSGCID pipeline. Since the beginning of the project, 2161 community requests have been received from 98 groups, of which 1384 have been approved and 1078 have entered into the SSGCID pipeline (see Table 2 ▶ and Fig. 3 ▶). 511 of the requests were received during the preparation of this manuscript and thus are still being processed for submission to NIAID and entry into the pipeline. Included in the community requests are 770 unique targets internally selected by SSGCID and subsequently requested by members of the scientific community. Consequently, these targets have been converted into community requests.

### 3.2. Structures solved   

All protein structures solved by SSGCID are submitted to the Protein Data Bank, while target status and protocols are submitted to the PSI TargetDB and PepcDB. The 318 structures submitted to the PDB by SSGCID include 75 structures from 38 different community-request targets (see Table 2 ▶). SSGCID structures provide a previously unavailable resource for researchers working on many pathogens, since they represent a substantial portion of all PDB entries for a number of genera. For example, SSGCID has solved 100% of all PDB entries for *Anaplasma* (ten), *Ehrlichia* (nine) and *Rickettsia* (six), 87% of all entries for *Babesia* (seven), 77% of all entries for *Brucella* (42), 68% of all entries for *Bartonella* (22) and 61% of all entries for *Coccidioides* (eight).

SSGCID works closely with members of the scientific community to publish protein structural data produced by the consortium and this has resulted in a number of collaborative publications (Yamada *et al.*, 2010 ▶; Edwards *et al.*, 2010 ▶; Jaffe *et al.*, 2011 ▶; Zhang *et al.*, 2011 ▶; Buchko *et al.*, 2010 ▶, 2011 ▶; Li *et al.*, 2010*a* ▶,*b* ▶; Moreno *et al.*, 2010 ▶).

### 3.3. SSGCID material resources   

Clones produced by the SSGCID are made available through the Biodefense and Emerging Infections Research Resources Repository (BEIR; http://www.beiresources.org/). To date, over 2600 clones are available for order and more are deposited each quarter. More than 1400 proteins (purified as single final peaks by size-exclusion chromatography in ∼10–150 mg quantities) produced by SSGCID can be ordered online through the SSGCID Protein Sample Distrbution System (SSGCID-PSDS). The PSDS site (http://www.ssgcidproteins.org) is partnered with Emerald BioSystems and will be accessible from the BEI Resources website by the fall of 2011. The only cost to the end-user for these proteins is a nominal charge to cover shipping on dry ice.

## 4. Future outlook   

At the time of writing, the SSGCID has submitted over 300 structures to the PDB from proteins encoded by bacteria, parasites and viruses causing human infectious disease. The current rate of solving structures is approximately two new depositions every week, putting us on track to exceed the project’s five-year goal. CSGID, the sister center to SSGCID, has solved structures at a similar pace. Thus, it is anticipated that together SSGCID and CSGID will submit over 1000 structures from infectious disease drug targets to the PDB by the end of the five-year contract period (late 2012). For many organisms this represents the vast majority of protein structures available and thus provides a heretofore unavailable opportunity for researchers to exploit structure-based drug-design approaches in order to develop novel chemotherapeutic agents against these diseases. SSGCID is committed to engaging the infectious disease research community in collaborations to maximize the potential for exploitation of the recent advances in structural genomics. The following articles in this special issue serve to communicate SSGCID’s progress and engender even more interest from the scientific community.

## 5. Overview of following papers   

This volume of *Acta Crystallographica Section F* represents a unique perspective on the SSGCID, as it is comprised of laboratory and structure communications prepared entirely by the scientists who work within the consortium itself. This volume contains several methodological papers that provide details of the high-throughput pipeline of the SSGCID: synthetic gene construction with *Gene Composer* software, fusion tags and cleavage methods for maximum yields from large-volume protein expression and specialized instrumentation for parallel protein purification and crystallization. Specifically, Choi and coworkers show that screening for IMAC recovery (immobilized metal-affinity chromatography) at early high-throughput screening and later large-scale expression screens help to identify the proteins that are most likely to be successful in upscaling, purification and crystal trials (Choi *et al.*, 2011 ▶). Additionally, Bryan and coworkers demonstrate that 3C protease cleavage improves the chances that a given protein will produce a structure (Bryan *et al.*, 2011 ▶). The structure communications in this volume cover a broad range of Category A, B and C pathogens, including both bacterial (*Rickettsia prowazekii*, *Ehrlichia chaffeensis* and *Burkholderia pseudomallei*) and eukaryotic (*Giardia lamblia*, *Coccidioides immitis*, *Babesia bovis* and *Cryptosporidium parvum*) pathogens, some of which represent one of very few protein structures available for the organism in the PDB. In many instances, these communications compare the apo structure of a protein with one or more ligand-bound complexes, including those produced through fragment screening or obtained using explicit transition-state mimetics. These comparative structural investigations, both in solution-state and crystal forms, now serve to enhance the understanding of the catalytic mechanisms of these targets and provide a basis for asking questions at the outset of rational structure-based drug-design research.

## Figures and Tables

**Table 1:** SSGCID success rates by taxon

Taxon	Cloned	Soluble (%)	Purified (%)	Crystals (%)	Diffraction (%)	Structure (%)	Overall (%)
**Bacteria**	**3383**	**57**	**60**	**51**	**51**	**66**	**6**
*Anaplasma*	162	51	72	29	53	100	6
*Bartonella*	222	64	68	46	71	66	9
*Borrelia*	161	48	61	49	39	56	3
*Brucella*	303	61	51	72	64	72	10
*Burkholderia*	780	54	38†	48	70	69	5†
*Ehrlichia*	120	72	64	44	58	64	8
*Mycobacterium*	1503	59	69	53	41	65	6
*Rickettsia*	104	47	69	47	50	50	4
Other genera‡	28	50	93	38	80	25	4
**Eukaryotes**	**758**	**56**	**71**	**44**	**43**	**61**	**5**
*Babesia*	28	61	82	71	60	83	18
*Coccidioides*	65	57	84	52	56	78	11
*Cryptosporidium*	75	55	68	32	33	33	1
*Encephalitozoon*	116	66	68	48	28	71	4
*Entamoeba*	245	49	70	39	58	53	4
*Giardia*	116	59	88	45	33	56	4
*Toxoplasma*	81	60	49	42	30	33	1
Other genera§	32	41	62	38	33	100	3
**Viruses**	**30**	**47**	**93**	**69**	**44**	**75**	**11**
*Filoviridae* §	5	20	0				0
*Orthomyxoviridae*	25	52	100	69	44	75	12
Other viruses	7	43	67	50	100	100	14
**Total**	**4178**	**57**	**62**	**50**	**50**	**66**	**6**

† The success rate for *Burkholderia* is artificially low, since purification of a large number of soluble targets has not yet been completed.

‡ Bacterial and eukaryotic genera with 25 or fewer targets are not shown individually.

§ Viruses are grouped by family.

**Table 2:** Summary of community-request targets in the SSGCID pipeline

Community-request targets	
Requestors	98
Requests received†	2161
Unique targets approved	1384
Unique targets, work started	1078
PDB submissions	
Total unique targets	38
Claimed by requestor before target solved	17
Claimed by requestor after target solved	21
Total unique structures	75
Claimed by requestor before target solved	27
Claimed by requestor after target solved	48

† Includes multiple requests for the same target.
